# Seam Carving Forgery Detection Through Multi-Perspective Explainable AI

**DOI:** 10.3390/jimaging11110416

**Published:** 2025-11-18

**Authors:** Miguel José das Neves, Felipe Rodrigues Perche Mahlow, Renato Dias de Souza, Paulo Roberto G. Hernandes, José Remo Ferreira Brega, Kelton Augusto Pontara da Costa

**Affiliations:** School of Sciences, São Paulo State University (UNESP), Bauru 17033-360, Brazil; f.mahlow@unesp.br (F.R.P.M.); renato.dias@unesp.br (R.D.d.S.); remo.brega@unesp.br (J.R.F.B.); kelton.costa@unesp.br (K.A.P.d.C.)

**Keywords:** explainable AI (XAI), seam carving, deep learning, image forensics, SHAP, convolutional neural network (CNN)

## Abstract

This paper addresses the critical challenge of detecting content-aware image manipulations, specifically focusing on seam carving forgery. While deep learning models, particularly Convolutional Neural Networks (CNNs), have shown promise in this area, their black-box nature limits their trustworthiness in high-stakes domains like digital forensics. To address this gap, we propose and validate a framework for interpretable forgery detection, termed E-XAI (Ensemble Explainable AI). Conceptually inspired by Ensemble Learning, our framework’s novelty lies not in combining predictive models, but in integrating a multi-perspective ensemble of explainability techniques. Specifically, we combine SHAP for fine-grained, pixel-level feature attribution with Grad-CAM for region-level localization to create a more robust and holistic interpretation of a single, custom-trained CNN’s decisions. Our approach is validated on a purpose-built, balanced, binary-class dataset of 10,300 images. The results demonstrate high classification performance on an unseen test set, with a 95% accuracy and a 99% precision for the forged class. Furthermore, we analyze the model’s robustness against JPEG compression, a common real-world perturbation. More importantly, the application of the E-XAI framework reveals how the model identifies subtle forgery artifacts, providing transparent, visual evidence for its decisions. This work contributes a robust end-to-end pipeline for interpretable image forgery detection, enhancing the trust and reliability of AI systems in information security.

## 1. Introduction

Explainable Artificial Intelligence (XAI) has become a critical field of research, particularly for understanding the decision-making processes of complex models like deep neural networks in tasks such as image classification [1]. In an era where such models are increasingly deployed in real-world, critical systems, the transparency of their internal workings is essential for establishing user trust, ensuring accountability, and facilitating adoption [2]. This need has spurred the development of a wide range of XAI techniques, from early efforts to visualize the features learned by deep networks [3] to contemporary methods like SHAP (Shapley Additive Explanations) for feature attribution and Grad-CAM for visualizing class-discriminative regions [4,5]. A significant challenge in digital forensics and computer vision lies in the detection of content-aware image manipulations. One such sophisticated technique is seam carving, an algorithm that intelligently resizes images by removing or inserting paths of low-energy pixels (seams), thereby preserving semantically important content [6]. While effective, this process introduces subtle, often imperceptible, structural artifacts. Although deep learning models, particularly Convolutional Neural Networks (CNNs), have shown promise in detecting such forgeries, their inherent black-box nature creates a critical gap. This lack of transparency limits their trustworthiness and utility as forensic tools, where understanding how a decision is made is as important as the decision itself. This work directly addresses this interpretability gap in forgery detection. We propose and validate a comprehensive framework, termed e-XAI (Ensemble Explainable AI), designed not only to classify images manipulated by seam carving with high accuracy but also to provide a deep, multi-perspective understanding of the model’s decision-making process. Our methodology is conceptually inspired by Ensemble Learning [7], yet its novelty lies not in combining multiple predictive models, but in creating an ensemble of explainability techniques. Specifically, we integrate SHAP for fine-grained, pixel-level attribution with Grad-CAM for region-level localization. This multi-perspective approach provides a more robust and holistic interpretation of a single predictive model’s behavior than either technique could offer alone. The proposed framework is validated using a purpose-built, balanced, binary-class dataset derived from a collection of images widely used in digital forensics research [8]. By demonstrating a complete end-to-end pipeline—from dataset creation and model training to a detailed, multi-faceted explainability analysis—this paper contributes a robust and transparent solution for detecting seam carving forgery. Ultimately, this work addresses the problem of opaque forgery detection by delivering a methodology that enhances both the performance and the trustworthiness of AI models in information security.

## 2. Related Works

The detection of content-aware image forgeries, particularly those created by the seam carving algorithm, has long been an active area of research in digital forensics. While early approaches relied on statistical analysis of image properties disrupted by the manipulation, the field has increasingly adopted deep learning. Specifically, Convolutional Neural Networks (CNNs) have proven effective due to their ability to automatically learn the subtle, high-dimensional artifacts left by such forgeries. Several studies have successfully designed custom CNN architectures to distinguish between original and seam-carved images, demonstrating high classification accuracy on controlled datasets [9,10]. Other works have explored more complex scenarios, such as detecting forgeries in compressed JPEG images, where compression artifacts can obscure the traces of manipulation [11,12]. Our work builds upon this strong foundation of using CNNs for forgery detection, but addresses their inherent lack of transparency.

Concurrently, the field of Explainable Artificial Intelligence (XAI) has grown in response to the black-box nature of many deep learning models. For high-stakes applications like digital forensics, a simple classification output (e.g., “forgery detected”) is often insufficient; an analyst must understand why the model arrived at that conclusion. Seminal guides and frameworks, such as those proposed by Molnar [13] and Doshi-Velez and Kim [2], have established the importance of interpretability for building trust and accountability in AI systems. Although XAI has been applied to various security domains like malware detection [14], its specific application to elucidate the detection of seam carving artifacts remains less explored. Our research confronts this gap directly, by not only detecting the forgery but also using XAI to visualize the specific artifacts upon which the model’s decision relies.

Furthermore, our methodology is conceptually inspired by Ensemble Learning, a technique renowned for improving the robustness and generalization of machine learning models [7]. However, while traditional ensembles combine multiple predictive models—as effectively demonstrated in contexts like big data stream learning [15]—our work proposes a novel interpretation: an ensemble of explainability techniques. By combining different XAI methods, such as SHAP for attribution and Activation Maximization for visualizing learned features [3], we aim to create a more comprehensive and reliable interpretation of a single, powerful predictive model. This approach contrasts with those that focus solely on predictive performance, providing a more holistic framework for building trustworthy forensic tools. This study, therefore, is positioned at the intersection of these three key areas: deep learning-based forgery detection, explainable AI, and ensemble-inspired methodologies, aiming to provide a solution that is both accurate and transparent.

## 3. Theoretical Basis

This paper is grounded on the intersection of three key domains: content-aware image forgery, deep learning for computer vision, and Explainable Artificial Intelligence (XAI). This section provides the necessary theoretical background for each of these areas, establishing the foundation for the methodology proposed.

### 3.1. Seam Carving Forgery

Seam carving is a sophisticated content-aware image resizing algorithm proposed by Avidan and Shamir [6]. Unlike standard scaling or cropping, it intelligently removes or inserts low-energy paths of pixels, known as “seams,” to alter image dimensions while preserving semantically important content. The energy of a pixel is often derived from its gradient magnitude, meaning textured or high-contrast regions are preserved while smoother areas are altered. From a digital forensics perspective, detecting seam carving is challenging because it introduces subtle, distributed artifacts rather than obvious localized inconsistencies. Identifying these subtle statistical disruptions is a task well-suited for deep learning models [9].

### 3.2. Convolutional Neural Networks (CNNs)

Convolutional Neural Networks (CNNs) are the state-of-the-art deep learning architecture for image analysis tasks [16]. Their design is inspired by the human visual cortex and consists of several core components:

- Convolutional Layers: Apply learnable filters to input images to detect specific features like edges, corners, and textures, creating feature maps.
- Activation Functions: Non-linear functions like the Rectified Linear Unit (ReLU) [17] are applied to enable the model to learn complex patterns.
- Pooling Layers: Downsample the feature maps to reduce computational complexity and create representations that are robust to small translations.
- Fully Connected Layers: Perform the final classification based on the high-level features extracted by the preceding layers.

This hierarchical structure allows CNNs to learn a rich set of features automatically, making them highly effective for tasks like image forgery detection.

### 3.3. Explainable Artificial Intelligence (XAI)

While powerful, the black-box nature of CNNs is a major barrier to their adoption in critical applications like forensics, where understanding the rationale behind a decision is crucial [1]. Explainable AI (XAI) aims to address this by providing insights into model behavior. Our work employs two complementary XAI techniques:

- SHAP (Shapley Additive Explanations): A game-theoretic approach that computes the contribution of each input feature (e.g., each pixel) to the model’s prediction [4]. It provides both local (per-image) and global (model-wide) explanations.
- Grad-CAM (Gradient-weighted Class Activation Mapping): A visualization technique that produces coarse localization maps highlighting the most important regions in an image for predicting a specific class [5]. It uses the gradients flowing into the final convolutional layer to identify which parts of an image were most influential, providing a high-level, interpretable view of where the model is looking.

Our proposed e-XAI framework, conceptually inspired by Ensemble Learning [7], combines these XAI methods to offer a more holistic and trustworthy interpretation of the forgery detection model.

## 4. Materials and Methods

**Figure 1:**
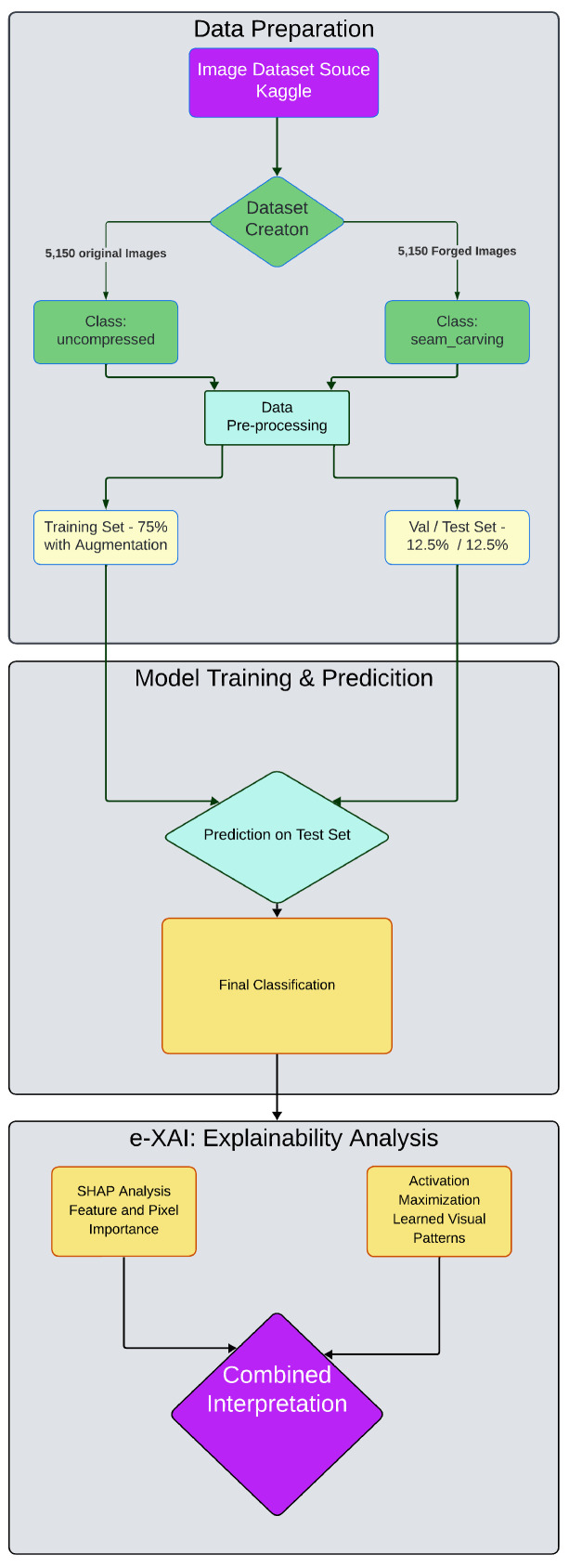
Overview of the proposed e-XAI workflow for seam carving detection.

This study proposes and evaluates a complete workflow for the detection of seam carving forgery in digital images, as schematically depicted in Figure 1. The methodology is centered on training a custom Convolutional Neural Network (CNN) and subsequently analyzing its decision-making process through a combined explainability framework, which we term Ensemble Explainable AI (e-XAI). The entire process, from data preparation to model training and interpretability analysis, is detailed in the following sections.

### 4.1. Dataset Construction

The dataset for this study was specifically constructed by the authors to create a controlled environment for detecting seam carving forgery. The process began with a base set of 5150 original, uncompressed images. These source images were obtained from a publicly available collection designed for digital image forensics [11,12], which contains images stored at different JPEG quality levels.

For each of the 5150 original images, a corresponding manipulated version was generated by applying the content-aware seam carving algorithm [6]. This algorithm intelligently removes vertical or horizontal seams of low-energy pixels to resize the image while preserving important content. This procedure resulted in a perfectly balanced, binary-class dataset comprising 10,300 images in total. The two classes are defined as follows:

- uncompressed: The 5150 original images. This class is referred to as uncompressed.
- seam carving doctored: The 5150 corresponding images that were resized using the seam carving removal process. This class is referred to as ‘seam_carving’.

### 4.2. Pre-Processing and Data Augmentation

The entire data pre-processing pipeline was implemented in Python (3.7.16) using libraries such as OpenCV and Scikit-learn. The primary goal was to prepare the image data for consumption by the deep learning model and to structure the training and evaluation process robustly.

#### 4.2.1. Image Standardization

Each image from the dataset was first loaded and converted from the BGR color space to RGB. Subsequently, all images were resized to a uniform dimension of 256×256 pixels. Finally, a normalization step was performed by scaling the pixel intensity values to the range [0,1] by dividing each pixel value by 255.0.

#### 4.2.2. Data Splitting

The complete dataset of 10,300 images was partitioned into three distinct, non-overlapping sets to ensure a robust evaluation: a training set (75%, 7725 images), a validation set (12.5%, 1287 images), and a final test set (12.5%, 1288 images). The split was performed using stratification to maintain an equal balance between the uncompressed and ‘seam_carving’ classes in all three sets. The training set was used to fit the model parameters, the validation set was used during training to monitor performance for early stopping and model selection, and the test set was held out exclusively for the final, unbiased evaluation of the model’s performance on unseen data.

#### 4.2.3. Data Augmentation

To prevent overfitting and improve model generalization, data augmentation was applied exclusively to the training set using Keras’ ‘ImageDataGenerator’ (developed by François Chollet and contributors, maintained by the TensorFlow team at Google, Mountain View, CA, USA). Real-time transformations included random rotations (up to 20 degrees), horizontal/vertical shifts (up to 20%), shear transformations, zooming (up to 20%), and random horizontal flipping.

### 4.3. CNN Architecture and Training

**Figure 2:**
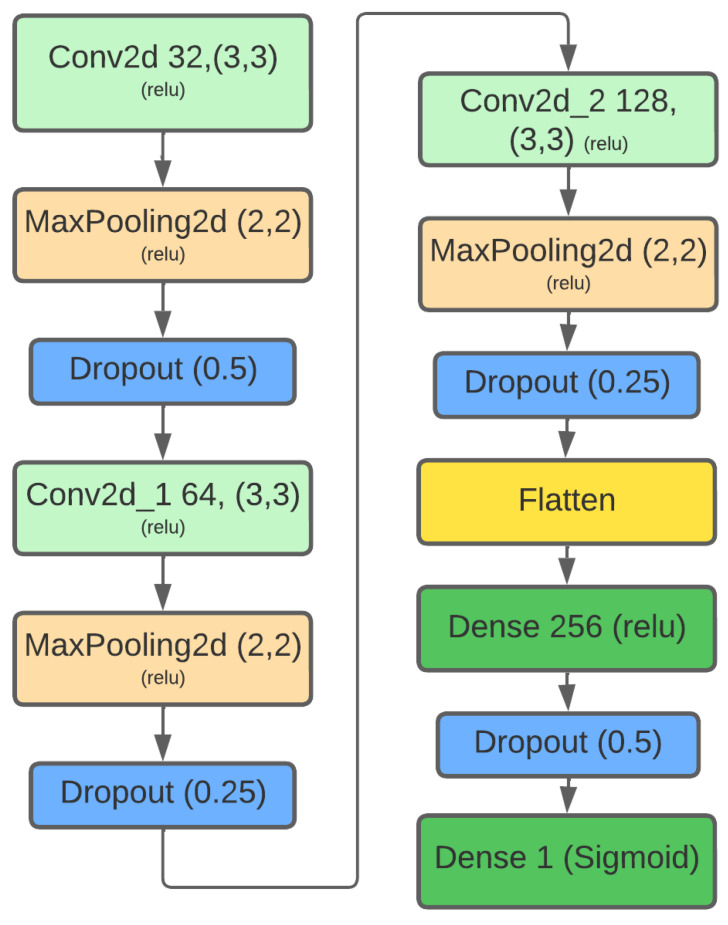
The custom Convolutional Neural Network architecture used for seam carving detection.

For the binary classification task, a custom CNN was designed and implemented using the Keras API within TensorFlow [18]. While pre-trained architectures such as VGGNet or ResNet excel at tasks requiring high-level semantic feature extraction, the detection of seam carving artifacts relies on identifying subtle, low-level statistical disturbances distributed across the image. These artifacts are non-semantic in nature and differ fundamentally from the object-centric features that pre-trained models are optimized to recognize. Consequently, a custom CNN architecture, trained from scratch on our purpose-built dataset, is more appropriate. This approach avoids the inherent bias of features learned from large-scale, general-purpose datasets like ImageNet and allows the model to learn the specific, fine-grained patterns indicative of seam carving forgery directly from the data. The model’s architecture, detailed in Figure 2, consists of the following sequential layers:

1. Three Convolutional Blocks: The architecture begins with three blocks, each comprising a Conv2D layer (with 32, 64, and 128 filters, respectively, all 3×3 with ReLU activation [17]), a 2×2 MaxPooling2D layer, and a Dropout layer (rate of 0.25). This design progressively increases feature complexity while reducing spatial dimensions, a common pattern inspired by pioneering architectures like VGGNet [19].
2. Classifier Head: The resulting feature maps are flattened and passed to a Dense layer with 256 neurons (ReLU activation) and a Dropout layer (rate of 0.5) for regularization.
3. Output Layer: A single Dense neuron with a Sigmoid activation function outputs a probability score between 0 and 1, representing the model’s confidence that the input image belongs to the ‘seam_carving’ class.

The model was compiled using the Adam optimizer with a learning rate of $1\times 10^{-4}$ and ‘binary_crossentropy’ as the loss function. Training was performed with a batch size of 32 for up to 100 epochs, utilizing ‘EarlyStopping’ (patience = 10 on ‘val_loss’) and ‘ModelCheckpoint’ (monitoring ‘val_accuracy’) callbacks to ensure robustness and prevent overfitting. Key hyperparameters are summarized in Table 1.

### 4.4. The e-XAI Methodology: A Combined Framework for Explainability

To move beyond a single perspective on model interpretability, we propose and apply a framework named Ensemble Explainable AI (e-XAI). This methodology is conceptually inspired by the principles of Ensemble Learning [7], where combining multiple diverse components often leads to a more robust outcome. It is important to note that our e-XAI framework does not ensemble multiple predictive models to improve accuracy. Instead, it assembles multiple, distinct explainability techniques to generate a more holistic and trustworthy understanding of a single, trained CNN model.

The core idea is to analyze the model’s behavior from complementary viewpoints, providing a multi-faceted explanation of its decisions. This multi-perspective approach combines a fine-grained, pixel-level attribution method (SHAP) with a broader, region-level localization method (Grad-CAM). While SHAP identifies precisely which pixels contribute most to a prediction, Grad-CAM highlights which regions of an image the model focuses on. Synthesizing these different “views” allows for a comprehensive interpretation: the model focuses on broad, structurally complex regions (identified by Grad-CAM), and within those regions, it detects specific pixel-level statistical artifacts (pinpointed by SHAP) to make its final decision. This synergy addresses a key objective of the e-XAI framework: to provide a deeper and more reliable understanding of the model’s decision-making process. The specific techniques are detailed below.

#### 4.4.1. Pixel-Level Attribution with SHAP

Shapley Additive Explanations (SHAP) is a game-theoretic approach used to explain the output of any machine learning model by quantifying the contribution of each feature to a prediction [4]. For image data, this translates to assigning an importance value to each pixel. We apply SHAP to generate visualizations that highlight the most influential pixels for the ‘seam_carving’ vs. uncompressed classification, revealing the specific, low-level evidence that led to the model’s decision. For example, after the model predicts an image as ’seam_carving’, SHAP analysis is applied. It computes an attribution map where each pixel is assigned a value. Pixels with high positive SHAP values, typically colored in red, are those that provided the strongest evidence for the ’seam_carving’ prediction, thus pinpointing the subtle artifacts the model detected.

#### 4.4.2. Region-Level Localization with Grad-CAM

Gradient-weighted Class Activation Mapping (Grad-CAM) is a visualization technique that produces coarse localization maps highlighting the most important regions in an image for predicting a specific class [5]. Unlike SHAP’s detailed pixel map, Grad-CAM provides a higher-level, more semantically interpretable view of where the model is “looking.” It identifies which parts of the image were most influential in the classification, often corresponding to areas with complex textures or structures where forgery artifacts are more likely to be found. For instance, Grad-CAM utilizes the gradients flowing into the final convolutional layer to produce a coarse heatmap. This heatmap highlights broad regions, such as areas of complex texture where the seam carving algorithm’s impact would be most pronounced, effectively answering the question of ’where’ the model is focusing its attention.

## 5. Results and Discussion

This section presents the performance evaluation of the custom-trained Convolutional Neural Network (CNN) for seam carving detection, followed by an in-depth analysis of its decision-making process using the proposed e-XAI framework. The results are organized into two main parts: quantitative performance metrics and qualitative insights from the explainability techniques.

### 5.1. Model Performance Evaluation

The model’s performance was rigorously evaluated on a dedicated, unseen test set of 1288 images, which maintained a perfect 50/50 balance between the uncompressed and ‘seam_carving’ classes. This balanced test set ensures that metrics like accuracy are meaningful and not skewed by class distribution.

#### 5.1.1. Classification Metrics and Confusion Matrix

The overall performance of the model is summarized in Table 2. The CNN achieved a high overall accuracy of 95.73%, indicating its strong capability to distinguish between original and manipulated images.

**Figure 3:**
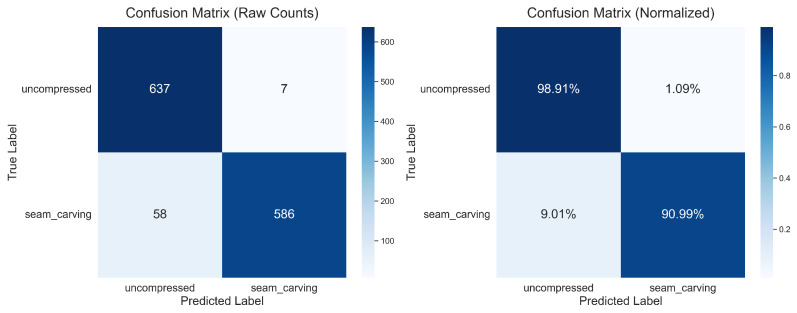
Confusion matrices for the CNN model on the test set. The matrix on the left displays the raw counts of predictions. The matrix on the right presents the normalized results, where each row is normalized by the true class total; the values on the diagonal therefore represent the recall (or True Positive Rate) for each class. This normalized view clearly shows a recall of 98.91% for the uncompressed class and 90.99% for the ‘seam_carving’ class.

A detailed breakdown of the classification results is provided by the confusion matrices in Figure 3. The model correctly identified 637 of the 644 ‘uncompressed’ images (True Negatives) and 586 of the 644 seam_carving images (True Positives). Notably, the model exhibited a very low number of False Positives (7, calculated from 644 to 637), resulting in a high precision of 99% for the seam_carving class. This implies that when the model flags an image as manipulated, it is highly likely to be correct, which is a critical feature for forensic applications. The main source of error was in False Negatives (58), where manipulated images were misclassified as original, suggesting that the artifacts in these specific instances were exceptionally subtle.

#### 5.1.2. ROC Curve and AUC

**Figure 4:**
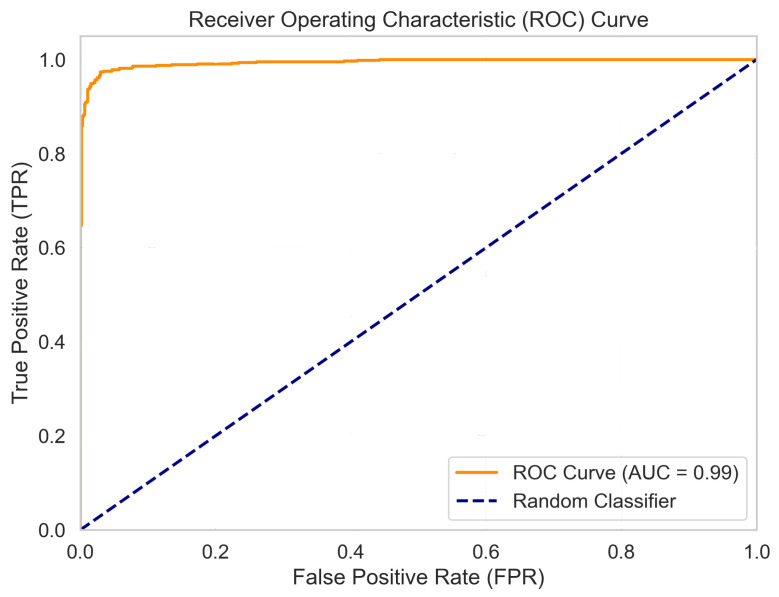
ROC curve for the CNN model on the test set. The high AUC score of 0.99 indicates excellent discriminative performance.

To assess the model’s discriminative ability across all decision thresholds, a Receiver Operating Characteristic (ROC) curve was plotted, as shown in Figure 4. The Area Under the Curve (AUC) was calculated to be 0.99, which is exceptionally close to a perfect classifier (AUC = 1.0). This high AUC value confirms that the model has an excellent capacity to distinguish between the two classes, independent of a specific classification threshold.

### 5.2. Insights from the e-XAI Framework

**Figure 5:**
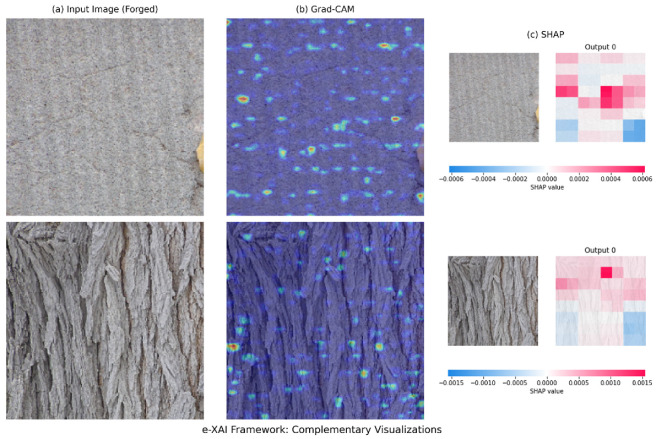
Complementary visualizations from the e-XAI framework for two forged images. (**a**) The original input image classified as ‘seam_carving’, shown without any color overlay. (**b**) Grad-CAM heatmap overlay, where warmer colors (red/yellow) indicate regions the model deemed most important. (**c**) SHAP attribution map, where red pixels positively contributed to the ‘seam_carving’ classification and blue pixels contributed to the uncompressed class.

Beyond quantitative performance, the e-XAI framework was applied to understand how the model achieves its classification results. By combining Grad-CAM for regionlevel localization and SHAP for pixel-level attribution, we can construct a comprehensive narrative of the model’s decision-making process. Figure 5 illustrates this multi-perspective analysis on representative test images classified as ‘seam_carving’.

The Grad-CAM heatmaps (column b) reveal that the model consistently focuses on areas with complex textures and structural content, such as the rocky ground in the top example or the patterned fabric in the bottom one. These are precisely the regions where the seam carving algorithm must make more intricate decisions to preserve visual coherence, thereby leaving more detectable statistical traces. Grad-CAM effectively answers the following question: “Where in the image did the model look for evidence?”

Complementing this high-level view, the SHAP attribution maps (column c) answer the following subsequent question: “What specific evidence did the model find there?” The SHAP values do not highlight semantically meaningful objects. Instead, the red pixels, indicating a positive contribution to the ‘seam_carving’ class, are often scattered within the high-attention regions identified by Grad-CAM. This pattern is consistent with the nature of seam carving artifacts: they are low-level, distributed statistical disturbances, not localized visual anomalies.

The synergy between the two techniques provides a robust and interpretable conclusion. The model first identifies regions susceptible to manipulation (via Grad-CAM) and then, within those regions, it pinpoints the subtle, pixel-level irregularities that confirm the presence of forgery (via SHAP). This multi-faceted explanation moves beyond a simple “forgery detected” output, offering transparent, visual evidence that is crucial for building trust in forensic applications.

#### 5.2.1. Quantitative Analysis of Explainability Maps

To statistically validate the behavior of the explainability maps, we investigated the hypothesis that the model’s attention, as visualized by Grad-CAM, correlates with the visual complexity of image regions. We quantified local image complexity using the variance of the Laplacian operator, a common metric for texture and edge detection. For a subset of test images, we computed the Pearson correlation coefficient between the mean Grad-CAM intensity and the Laplacian variance across a grid of non-overlapping image patches.

The analysis revealed a statistically significant positive correlation (*r* = 0.3220, *p* < 0.001). This result quantitatively confirms that the CNN directs its focus towards regions of higher textural complexity, which are precisely the areas where the seam carving algorithm is forced to make more intricate decisions, thus leaving more detectable artifacts. This analysis provides statistical support for the qualitative insights gathered from the XAI visualizations.

#### 5.2.2. Robustness Analysis Against JPEG Compression

To assess the model’s robustness in real-world scenarios where images may undergo additional processing, we evaluated its performance against varying levels of JPEG compression. The original test set images were recompressed at JPEG quality levels of 90, 70, and 50. The trained model was then evaluated on each of these newly compressed test sets.

The results, summarized in Table 3, demonstrate a clear degradation in performance as the JPEG compression level increases. The accuracy dropped from 95.7% on the original test set to 93.7% at quality 90, 78.1% at quality 70, and finally to 61.8% at quality 50. This behavior is expected, as strong JPEG compression is known to introduce high-frequency artifacts that can obscure or destroy the subtle traces left by the seam carving algorithm. This analysis highlights a limitation of the current model and underscores that its effectiveness is highest on uncompressed or lightly compressed images. It also suggests that including compressed images during data augmentation could be a key strategy for enhancing the model’s robustness in future work.

### 5.3. Comparison with State-of-the-Art Methods

To contextualize the performance of our proposed model, we compare our results with several state-of-the-art (SOTA) deep learning-based methods for seam carving detection. While a direct, one-to-one comparison is challenging due to variations in datasets and experimental protocols, this assessment highlights the competitiveness of our approach. Table 4 summarizes the performance of our model against key works in the field.

As shown in Table 4, our custom-trained CNN achieves an accuracy of 95%, a result that is highly competitive and directly in line with state-of-the-art performance for this task. Specialized architectures, such as those proposed by Nam et al. [9] and Ye et al. [10], report slightly higher accuracies on their specific uncompressed datasets. Our model’s performance is also comparable to detectors evaluated on lightly compressed images, as seen in the study by Celebi et al. [12].

The primary contribution and novelty of our work, however, extend beyond classification accuracy. While these SOTA methods focus primarily on achieving high detection rates, our e-XAI framework provides a crucial additional layer of interpretability. Our approach not only achieves competitive accuracy but also delivers transparent, visual evidence of the model’s decision-making process through the synergistic use of SHAP and Grad-CAM. This focus on explainability addresses a critical gap in the literature, enhancing the trustworthiness and practical utility of the detection model for real-world forensic applications, where justifying a finding is as important as the finding itself. Therefore, our work presents a more holistic solution that successfully balances high performance with essential interpretability.

### 5.4. Computational Cost and Feasibility

To assess the practical applicability of the proposed method, we evaluated its computational cost. The custom CNN architecture comprises a total of approximately 33.6 million trainable parameters. The training process was conducted on a machine equipped with an NVIDIA RTX 3080 GPU, taking approximately 4 h to complete the training until the early stopping criterion was met. For deployment in a forensic workflow, the inference speed is a critical metric. On the same hardware, the model is capable of processing a single 256 × 256 image in approximately 15 milliseconds, making it suitable for analyzing large batches of images in a time-efficient manner.

## 6. Conclusions

This research introduced and validated a comprehensive methodology for detecting seam carving image forgery. By training a custom-built Convolutional Neural Network (CNN) on a purpose-built, balanced dataset, we achieved high classification performance, including a 95% accuracy and, critically for forensic applications, a 99% precision for the forged class on the dedicated, unseen test set. The central contribution of this work, however, lies in the application of our proposed Ensemble Explainable AI (e-XAI) framework. This framework, conceptually inspired by Ensemble Learning [7], does not combine predictive models but rather integrates distinct explainability techniques—SHAP for feature attribution and Grad-CAM for region-level localization. This dual approach provided a multi-faceted and deeper understanding of the CNN’s decision-making process.

Our results demonstrated that the trained CNN successfully learned to identify the subtle, distributed artifacts introduced by the seam carving algorithm. The XAI analysis revealed key insights into this process: SHAP visualizations highlighted that the model’s decisions were driven by specific, low-level pixel regions with statistical irregularities, rather than high-level semantic content. Complementarily, Grad-CAM highlighted the regions most influential for classification, providing a spatial perspective that complements SHAP’s pixel-level attribution. This dual-perspective explanation increases the transparency and trustworthiness of the black-box model, which is crucial for its adoption in digital forensics and information security.

The central challenge of detecting content-aware manipulations was effectively addressed. Our methodology confirms that even when alterations are not easily discernible to a human observer, a well-trained CNN can identify the underlying structural changes. The development of a specific, balanced dataset for this task was a necessary and important step, addressing a common bottleneck in the field of image forgery detection.

The findings have direct implications for information security, providing a robust and, most importantly, interpretable tool for identifying image alterations. Future work could expand upon this foundation in several ways: conducting detailed ablation studies to quantify the contribution of each architectural component (e.g., dropout rates, number of convolutional blocks); exploring more advanced deep learning architectures, such as transformers or attention-based models; investigating the model’s robustness against different JPEG compression levels not used in the training set; and developing the e-XAI framework into a more interactive tool for forensic analysts. Furthermore, applying this framework to other forms of subtle, content-aware manipulations would be a valuable next step.

In conclusion, this work presents a complete end-to-end pipeline for creating, training, and interpreting a deep learning model for seam carving detection. The proposed e-XAI methodology offers a viable path toward building more transparent and reliable AI systems for security applications, paving the way for further research in automated and, crucially, understandable image forgery detection.

## Figures and Tables

**Table 1:** Key hyperparameters for the CNN model.

Hyperparameter	Value
Input Image Size	256×256×3
Optimizer	Adam
Learning Rate	$1\times 10^{-4}$
Loss Function	Binary Cross-entropy
Batch Size	32
Max Epochs	100
Early Stopping Patience	10
Dropout Rates	0.25 (conv. blocks), 0.5 (dense layer)

**Table 2:** Classification report for the CNN model on the unseen test set.

Class	Precision	Recall	F1-Score	Support
uncompressed	0.92	0.99	0.95	644
seam_carving	0.99	0.91	0.95	644
Weighted Avg	0.95	0.95	0.95	1288

**Table 3:** Model accuracy vs. JPEG compression quality.

JPEG Quality Level	Test Set Accuracy
100 (Original)	95.73%
90	93.71%
70	78.11%
50	61.80%

Note: The table shows the degradation in model accuracy as the JPEG compression level applied to the test set increases (lower quality values mean higher compression).

**Table 4:** Performance comparison with state-of-the-art (SOTA) seam carving detection methods.

Method	Dataset Type	Reported Accuracy
Nam et al. (2019) [9]	Custom/Uncompressed	98.81%
Ye et al. (2019) [10]	Custom/Uncompressed	97.80%
Celebi et al. (2022) [12]	JPEG Compressed (QF95)	94–96%
Our Proposed Model	Custom/Balanced	approximately 96%

Note: Accuracy values for SOTA methods are based on results reported in their respective papers and may not be directly comparable due to different test conditions. QF refers to JPEG quality factor.

## Data Availability

The original data presented in the study are openly available in a GitHub repository at https://github.com/migmiguel29/SeamCarvingeXai (accessed on 12 August 2025).

## Abbreviations

The following abbreviations are used in this manuscript:

AI	Artificial Intelligence
AUC	Area Under the Curve
CNN	Convolutional Neural Network
e-XAI	Ensemble Explainable Artificial Intelligence
Grad-CAM	Gradient-weighted Class Activation Mapping
ReLU	Rectified Linear Unit
ROC	Receiver Operating Characteristic
SHAP	Shapley Additive Explanations
XAI	Explainable Artificial Intelligence
